# The acceptability and cost of a home-based chlamydia retesting strategy: findings from the REACT randomised controlled trial

**DOI:** 10.1186/s12889-016-2727-4

**Published:** 2016-01-28

**Authors:** K. S. Smith, J. M. Kaldor, J. S. Hocking, M. S. Jamil, A. M. McNulty, P. Read, C. S. Bradshaw, M. Y. Chen, C. K. Fairley, H. Wand, K. Worthington, S. Blake, V. Knight, W. Rawlinson, M. Saville, S. N. Tabrizi, S. M. Garland, B. Donovan, R. Guy

**Affiliations:** 1 The Kirby Institute, UNSW Australia, Sydney, Australia; 2 Melbourne School of Population and Global Health, University of Melbourne, Melbourne, Australia; 3 Sydney Sexual Health Centre, Sydney, Australia; 4 School of Public Health and Community Medicine, UNSW Australia, Sydney, Australia; 5 Kirketon Road Centre, Sydney, Australia; 6 Melbourne Sexual Health Centre, Melbourne, Australia; 7 Central Clinical School, Monash University, Melbourne, Australia; 8 Serology and Virology Division, (SAViD) SEALS Microbiology, Prince of Wales Hospital, Sydney, Australia; 9 VCS Pathology, Melbourne, Australia; 10 Department of Obstetrics and Gynaecology, University of Melbourne, Melbourne, Australia; 11 Department of Microbiology, Royal Children’s Hospital, Melbourne, Australia; 12 Department of Microbiology and Infectious Diseases, Royal Women’s Hospital, Melbourne, Australia; 13 Murdoch Childrens Research Institute, Melbourne, Australia

**Keywords:** Chlamydia, Retesting, Randomised controlled trial, Cost, Acceptability, Repeat infection, Home testing

## Abstract

**Background:**

Chlamydia retesting three months after treatment is recommended to detect reinfections, but retesting rates are typically low. The REACT (retest after *Chlamydia trachomatis*) randomised trial demonstrated that home-based retesting using postal home-collection kits and SMS reminders, resulted in substantial improvements in retesting rates in women, heterosexual men and men who have sex with men (MSM), with detection of more repeat positive tests compared with SMS reminder alone. In the context of this trial, the acceptability of the home-based strategy was evaluated and the costs of the two strategies were compared.

**Methods:**

REACT participants (200 women, 200 heterosexual men, 200 MSM) were asked to complete an online survey that included home-testing acceptability and preferred methods of retesting. The demographics, sexual behaviour and acceptability of home collection were compared between those preferring home-testing versus clinic-based retesting or no preference, using a chi-square test. The costs to the health system of the clinic-based and home retesting strategies and the cost per infection for each were also compared.

**Results:**

Overall 445/600 (74 %) participants completed the survey; 236/445 from the home-testing arm, and 141 of these (60 %) retested at home. The majority of home arm retesters were comfortable having the kit posted to their home (86 %); found it easy to follow the instructions and collect the specimens (96 %); were confident they had collected the specimens correctly (90 %); and reported no problems (70 %). Most (65 %) preferred home retesting, 21 % had no preference and 14 % preferred clinic retesting. Comparing those with a preference for home testing to those who didn’t, there were significant differences in being comfortable having a kit sent to their home (*p* = 0.045); not having been diagnosed with chlamydia previously (*p* = 0.030); and living with friends (*p* = 0.034). The overall cost for the home retest pathway was $154 (AUD), compared to $169 for the clinic-based retesting pathway and the cost per repeat infection detected was $1409 vs $3133.

**Conclusions:**

Among individuals initially diagnosed with chlamydia in a sexual health clinic setting, home-based retesting was shown to be highly acceptable, preferred by most participants, and cost-efficient. However some clients preferred clinic-based testing, often due to confidentiality concerns in their home environment. Both options should be provided to maximise retesting rates.

**Trial registration:**

The trial was registered with the Australia New Zealand Clinical Trials Registry on September 9, 2011: ACTRN12611000968976.

**Electronic supplementary material:**

The online version of this article (doi:10.1186/s12889-016-2727-4) contains supplementary material, which is available to authorized users.

## Background

*Chlamydia trachomatis* is the most commonly reported bacterial sexually transmissible infection (STI) in most developed countries and reported diagnoses continue to increase [1–3]. Repeat infection with chlamydia is also common. Prospective cohort studies have demonstrated that within 4–5 months, 20–30 % of young women have a repeat infection [4, 5]. Routine retesting data show repeat infections are also high in men who have sex with men (MSM) [6]. Repeat chlamydial infections increase the risk of chlamydia-related sequelae such as pelvic inflammatory disease and infertility, when compared with initial infection [7]. Repeat infections have been associated with increased risk of HIV transmission [8].

Clinical guidelines in several countries recommend retesting three months after treatment for chlamydia to detect reinfections [9–13], however retesting rates are typically low especially amongst men [14–16]. A key barrier for patients is the time and effort involved in returning to the clinic for retesting [17]. A range of single interventions (reminders or home-collection) aimed at increasing rescreening rates for chlamydial infection have been trialled but with modest impact [18–23]. Few studies have evaluated multi-faceted interventions or given people a choice of retesting options [17, 24, 25].

In 2011–2013, we undertook a randomised trial known as REACT (retest after *Chlamydia trachomatis*), to determine if the combination of a postal home-collection kit and short message service (SMS) reminder at three months would increase the proportion of sexual health clinic patients retesting for chlamydia at 1–4 months, compared to clinic-based retesting and SMS reminder. SMS reminders were standard practice at the clinics at the time of the study. Those in the home arm were also given a choice of returning to the clinic if they preferred. Overall 61 % of participants randomised to the home arm retested within 1–4 months of chlamydia diagnosis compared with 39 % randomised to the clinic arm (*p* < 0.01). There were also significantly more repeat positive tests detected in the home arm compared with the clinic arm (31 cases vs 12 cases, *p* < 0.01). Among participants in the home arm who retested at 1–4 months, 27 % (50/184) chose to retest at the clinic. In the context of this trial we evaluated the acceptability of the home-based retesting strategy to the patient, and compared the costs between the home-based strategy and routine clinic retesting. These were secondary outcomes of the REACT trial.

## Methods

### REACT trial methods

The REACT trial methods are described in detail elsewhere [26]. Briefly, in this unblinded RCT, individuals were randomised in a 1:1 ratio to either an SMS reminder and postal home-collection kit (intervention–home arm), or an SMS reminder and clinic testing (control–clinic arm). Participants in the home arm had the option to return to the clinic for retesting if they preferred. The trial was conducted in two public sexual health clinics in Australia (Melbourne and Sydney Sexual Health Centres). Participants included three risk groups: women, heterosexual men and MSM. To be eligible participants had to be: aged 16 years or above; with a diagnosis by nucleic acid amplification tests (NAAT) of chlamydial infection (genital infection in heterosexual men and women, and urethral and/or rectal infection in MSM); and reside in the jurisdiction serviced by the clinic for the next six months. They were also required to have a mobile phone. Patients were excluded if: they were unwilling or unable to comply with all the requirements of the protocol; they could not speak English; or were HIV positive or a current sex worker. Eligible patients were asked by study nurses for their permission to pass on their contact details to a member of the research team. If the patient agreed, a member of the research team contacted the patient to explain the trial requirements and undertake a verbal consent process as per ethics committee approval.

For participants in the home arm, 3 months after chlamydia diagnosis, an SMS was sent by the research team to let the patient know their retest was due and a kit would soon be mailed to them. The home collection kit contained the collection device/s (women, self-collected vaginal swab; heterosexual men, UriSwab for urine collection (Copan Diagnostics, Murrieta, CA); MSM, UriSwab and rectal swab), plus illustrated collection instructions, a laboratory request form, and a prepaid envelope. The swabs and request form were pre-labelled with identifying information. The collection kit was mailed to the patient in an unmarked package by the research team at 3 months. Patients were instructed in a covering letter to collect their specimen/s, package them according to the provided instructions, and mail them to the laboratory in the prepaid envelope.

For participants in the clinic arm, 3 months after chlamydia diagnosis, patients were sent an SMS reminder by the clinic to remind them to return to the clinic for retesting. This is routine practice at the two participating clinics.

The main outcome measures were the percentage of participants retested at 1–4 months after chlamydia diagnosis and the percentage in each arm with repeat positive tests, by risk group and overall, analysed by intention to treat, and these have been recently reported [27]. The focus of this paper is on the secondary outcomes which include: the acceptability of home-based retesting for chlamydia and SMS reminders; and the cost of home versus clinic-based retesting. These are described in detail below.

### Acceptability and cost analysis methods

#### Acceptability

REACT participants (200 women, 200 heterosexual men, 200 MSM) were sent an SMS 4.5 months after enrolment (after ascertainment of the primary outcome), asking them to complete an online survey (Additional file 1). The SMS contained the study website and the participant’s code which was linked to their patient details captured at consent. The survey captured: participant demographics; living situation (with parents or others); whether the participant retested and if not, reasons for not retesting; and the acceptability of SMS reminders (all participants). Those randomised to the home arm were also asked about their level of comfort with having a home test kit mailed to them, their ease and level of confidence with self-collection of specimens, problems with specimen collection, preferred methods of retesting in the future (home, clinic or either) and their preferred method of receiving home test kits in the future. The majority of questions used a Likert scale format to measure the participant’s response, or a choice of responses plus free-text space was provided for some questions. For the following questions: reason for not retesting, problems with specimen collection and reasons for retesting preference; participants could choose more than one option. Participants who did not complete the survey were sent a second SMS two weeks later. If the survey was still not completed within another two weeks, they were sent a third SMS asking if they would prefer the survey to be mailed to them or to complete it by telephone. On completion of the survey, participants were sent a $40 AUD voucher, irrespective of retesting.

The following clinical variables collected at the initial screening consultation were extracted from the clinic’s patient management system and linked with the survey results using the study identifier: date of birth, country of birth, condom use in the last 3 months (consistent, inconsistent, or never), number of sexual partners in the last 3 months, previous chlamydia diagnoses (ever), and anal and urogenital symptoms.

A chi-square test was used to assess if there were any differences in baseline characteristics: risk group, demographics, sexual behaviour or STI clinical information (previous chlamydia diagnosis or STI symptoms), between survey respondents in the home versus the clinic arm. A chi-square test was also used to assess if there were any differences in demographics, sexual behaviour and acceptability of home testing according to the preferred method of retesting (home versus clinic/ no preference).

#### Cost analysis

**Fig. 1:**
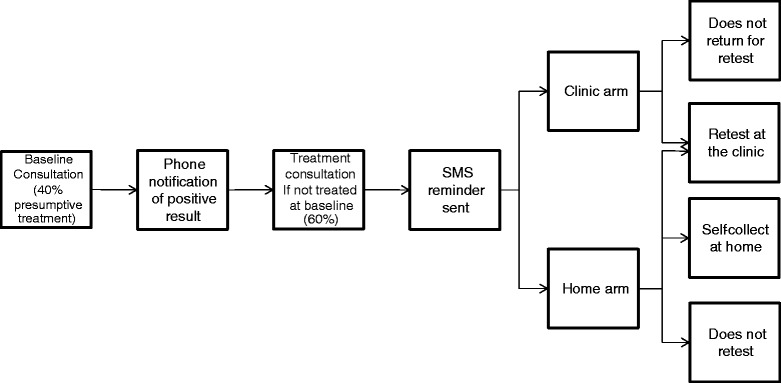
Flowchart of retest pathway

We assessed the cost of the chlamydia home retesting intervention compared to clinic based retesting from the perspective of the health system only. Based on routine practice at the participating sexual health services and the home-testing pathway used in the trial, a flowchart was constructed including all the steps from the initial chlamydia test to the retest, in both the clinic-based strategy and the home strategy (initial consultation, notification of a positive result, treatment consultation, SMS, retest) (Fig. 1). Based on information provided by the participating clinics, we assumed that 40 % of patients were treated at the initial consultation on the basis of symptoms or as contacts, and didn’t require a further treatment visit.

### Clinic costs

The two participating clinics conducted an audit of the last 20 clients who retested in each of the three risk groups, and provided the length of the consultation and staff type seen during the screening, treatment, and retesting visits. We then calculated the average consultation length for each risk group at each step. Additional information sourced from the clinics included the cost of SMS reminders, equipment, antibiotic treatment and telephone calls to inform clients of their results. Labour costs were estimated based on the length of consultation time, staff type and average salary including on-costs (25 %) and infrastructure (30 %). The cost of diagnostic testing was based on the fees charged by the laboratories (which included the cost of the test as well as the testing equipment, the request form, administration, courier and infrastructure). Using the above data, the clinical and laboratory costs of the entire testing pathway including registration, screening, treatment and retesting for clinic and home based testing was calculated.

### Home collection costs

The home collection costs were the same as for the clinic arm up to the retesting step. Costs for the home retest step included the cost of assembling the kit (based on the length of time taken and the research team member’s salary including on-costs and infrastructure as above), equipment additional to that which was supplied by the laboratory (saline for rectal swabs and postal satchels), return postage and the laboratory fee (which included the cost of the test, testing equipment, request form and administration).

### Overall costs and cost per infection detected

Based on the costs above, the costs of the entire clinic pathway versus the home test strategy were calculated. We also calculated the expected costs of clinic and home testing based on the uptake of retesting in each arm of the REACT trial, taking into consideration that 27 % of participants in the home arm chose to retest at the clinic. Based on these costs and repeat infections detected in each arm, the cost per infection detected for clinic and home testing was calculated by dividing the total costs for all participants in each arm by the number of infections detected.

### Ethics approval

This study was approved by the Alfred Health Human Research Ethics Committee (HREC), South Eastern Sydney and Illawarra Area Health Service HREC and the University of New South Wales HREC.

## Results

### Acceptability

#### Survey respondents

Overall 445/600 (74 %) of participants completed the survey (MSM 165/200 [83 %], women 150/200 [75 %], heterosexual men 130/200 [65 %]). Comparing survey respondents with non-respondents, there were significant differences in risk group and demographics, with a higher proportion of MSM and a lower proportion of heterosexual men (*p* = 0.001), and a lower proportion aged <30 years (*p* = 0.015). Overall 236 of 302 (78 %) home-arm participants and 209 of 298 (70 %) clinic arm participants completed the survey. There were no significant differences in baseline characteristics (risk group, demographics, sexual behaviour or STI clinical information) between survey respondents in the home versus the clinic arm (Table 1).

**Table 1:** Characteristics of survey respondents by study arm

Baseline characteristics	Overall	Clinic arm	Home arm	p-value^a^
	n (%)	n (%)	n (%)	
All	445 (100)	209 (47.0)	236 (53.0)	
Age group				0.480
< 30	294 (66.1)	137 (65.6)	157 (66.5)	
30–39	111 (24.9)	54 (25.8)	57 (24.2)	
40–49	23 (5.2)	8 (3.8)	15 (6.4)	
50+	17 (3.8)	10 (4.8)	7 (3.0)	
Risk group				0.491
Women	150 (33.7)	71 (34.0)	79 (33.5)	
Heterosexual men	130 (29.2)	56 (26.8)	74 (31.4)	
MSM	165 (37.1)	82 (39.2)	83 (35.2)	
Who they live with?				0.233
Partner	65 (14.6)	31 (14.8)	34 (14.4)	
Friends / flatmates	237 (53.3)	109 (52.2)	128 (54.2)	
Parents	49 (11.0)	26 (12.4)	23 (9.7)	
Alone	82 (18.4)	41 (19.6)	41 (17.4)	
Other	12 (3.0)	2 (1.0)	10 (4.2)	
Country of birth				0.870
Australia	215 (54.8)	103 (55.4)	112 (54.4)	
Asia	48 (12.2)	22 (11.8)	26 (12.6)	
Europe	118 (30.1)	55 (29.6)	63 (30.6)	
Other	11 (2.9)	6 (3.2)	5 (2.4)	
Previous CT diagnosis				0.841
Yes	56 (12.6)	27 (12.9)	29 (12.3)	
No	389 (87.4)	182 (87.1)	207 (87.7)	
Condom use in last 3 months (pre-diagnosis)				0.432
Inconsistent (never/ sometimes)	333 (81.8)	162 (84.4)	171 (79.5)	
Consistent (always)	74 (18.2)	30 (15.6)	44 (20.5)	
Number of sexual partners last 3 months				0.397
0	26 (5.9)	10 (4.8)	16 (6.8)	
1	117 (26.3)	62 (29.8)	55 (23.3)	
2–5	221 (49.8)	101 (48.6)	120 (50.8)	
> 5	80 (18.0)	35 (16.8)	45 (19.1)	
STI symptoms^b^ at baseline				0.503
Yes	196 (44.0)	96 (45.9)	100 (42.4)	
No	249 (56.0)	113 (54.1)	136 (57.6)	

Note: Data for some variables were not available for all participants

*MSM* men who have sex with men, *CT* Chlamydia trachomatis

^a^Chi^2^ or Fisher’s exact test was used when appropriate

^b^Anal and/or urogenital symptoms

#### Acceptability of SMS reminders (all participants)

The majority of survey respondents were comfortable or very comfortable with receiving an SMS reminder about retesting (85 %, *n* = 378) and were comfortable with the timing and wording of the SMS reminders (90 %, *n* = 399). Among those who nominated a single choice of their preferred reminder method (*n* = 401), 75 % had a preference for SMS and 15 % said they would prefer email reminders. Responses were similar between the home and clinic groups.

#### Reasons for not retesting (all participants)

Amongst the 28 survey respondents in the home arm who didn’t retest, the main reasons were: lack of time (32 %, *n* = 9), not considering themselves to be at risk (18 %, *n* = 5), misplacing their kit (14 %, *n* = 4), not receiving their kit (11 %, *n* = 3) and not being sure about how to self-collect specimens (7 %, *n* = 2). Among clinic arm respondents who didn’t retest (*n* = 67), the main reasons were: lack of time (43 %, *n* = 29), not considering themselves to be at risk (16 %, *n* = 11), no reminder (7 %, *n* = 5) and went overseas/ changed address (7 %, *n* = 5).

#### Acceptability of home collection (home arm participants only)

Of the participants from the home arm who completed the survey (*n* = 236), 141 (60 %) retested at home at any time during the study period (home-based retesters) (52 females, 43 heterosexual males and 46 MSM). The majority of home-based retesters were comfortable or very comfortable having the kit posted to their home (86 %, *n* = 121); found it easy to follow the instructions and collect the specimens (96 % both, *n* = 136 and 135 respectively); were confident they had collected the specimens correctly (90 %, *n* = 127); and reported no problems collecting the specimen (69 %, *n* = 97) (Table 2). Amongst those who reported problems collecting their specimens (*n* = 42), the main problems were not knowing how far to insert the swab (60 %, *n* = 25), urine splashing on the hands (26 %, *n* = 11), followed by difficulty aiming urine onto the swab (5 %, *n* = 2).

**Table 2:** Characteristics of participants who preferred home testing compared with clinic-based retesting or no preference

Baseline characteristics and survey responses	Overall	Prefer clinic or no preference	Prefer home testing	p-value^a^
	n (%)	n (%)	n (%)	
All	141 (100)	49 (34.8)	92 (65.2)	
Age group (years)				0.212
< 30	92 (65.3)	28 (57.1)	64 (69.6)	
30–39	34 (24.1)	17 (34.7)	17 (18.5)	
40–49	8 (5.7)	2 (4.1)	6 (6.5)	
≥ 50	7 (5.0)	2 (4.1)	5 (5.4)	
Risk group				0.916
Women	49 (34.8)	18 (36.7)	31 (33.7)	
Heterosexual men	43 (30.5)	14 (28.6)	29 (31.5)	
MSM	49 (34.8)	17 (34.7)	32 (34.8)	
Living arrangements				0.034
Friends/flatmates	75 (53.2)	18 (36.7)	57 (62.0)	
Alone	28 (19.9)	13 (26.5)	15 (16.3)	
Partner	19 (13.5)	9 (18.4)	10 (10.9)	
Parents	15 (10.6)	6 (12.2)	9 (9.8)	
Other	4 (2.8)	3 (6.1)	1 (1.1)	
Country of birth				0.980
Australia	74 (52.5)	25 (51.0)	49 (53.3)	
Europe	38 (27.0)	13 (26.5)	25 (27.2)	
Asia	10 (7.1)	4 (8.2)	6 (6.5)	
Other	16 (11.4)	6 (12.2)	10 (10.9)	
Previous CT diagnosis				0.030
Yes	15 (10.6)	9 (18.4)	6 (6.5)	
No	126 (89.4)	40 (81.6)	86 (93.5)	
Condom use last 3 months				0.809
Inconsistent (never/sometimes)	106 (75.2)	38 (77.6)	68 (73.9)	
Consistent (always)	27 (19.2)	8 (16.3)	19 (20.7)	
Missing	8 (5.7)	3 (6.1)	5 (5.4)	
Number of sexual partners last 3 months				0.137
None	5 (3.6)	1 (2.0)	4 (4.4)	
1	36 (25.5)	16 (32.7)	20 (21.7)	
2–5	74 (52.5)	20 (40.8)	54 (58.7)	
> 5	26 (18.4)	12 (24.5)	14 (15.2)	
STI symptoms^b^ at baseline				0.678
Yes	58 (41.1)	19 (38.8)	39 (42.4)	
No	83 (58.9)	30 (61.2)	53 (57.6)	
Baseline chlamydia specimen collection				0.905
Specimens collected by a clinician	93 (66.0)	32 (65.3)	61 (66.3)	
Self-collected specimens	48 (34.0)	17 (34.7)	31 (33.7)	
Comfortable having kit posted to home				0.045
Very comfortable	72 (51.1)	19 (38.8)	53 (57.6)	
Comfortable	49 (34.8)	19 (38.8)	30 (32.6)	
Neutral/uncomfortable	20 (14.2)	11 (22.5)	9 (9.8)	
How easy to understand instructions				0.948
Very easy	92 (65.3)	31 (63.3)	61 (66.3)	
Easy	44 (31.2)	16 (32.7)	28 (30.4)	
Neutral/hard	5 (3.6)	2 (4.1)	3 (3.3)	
How easy to collect specimen				0.259
Very easy	89 (63.1)	29 (59.2)	60 (65.2)	
Easy	46 (32.6)	16 (32.7)	30 (32.6)	
Neutral/hard	6 (4.3)	4 (8.2)	2 (2.2)	
How confident specimen collected correctly				0.450
Very confident	60 (42.6)	20 (40.8)	40 (43.5)	
Reasonably confident	67 (47.5)	22 (44.9)	45 (48.9)	
Neutral/not confident	14 (9.9)	7 (14.3)	7 (7.6)	
Any problems collecting the specimens				0.559
No	97 (68.8)	35 (71.4)	62 (67.4)	
Yes	42 (29.8)	13 (26.5)	29 (31.5)	
Comfort with SMS reminders				0.577
Comfortable/Very comfortable	126 (89.4)	45 ((91.8)	81 (88.0)	
Neutral/uncomfortable	15 (10.6)	4 (8.2)	11 (12.0)	

*MSM* men who have sex with men, *CT* Chlamydia trachomatis

^a^Chi^2^ or Fisher’s exact test was used when appropriate

^b^anal and/or urogenital symptoms

#### Preferred retesting strategy (home arm participants only)

The majority of home-based retesters (65 %, *n* = 92) said they would prefer home testing in the future, 21 % had no preference and 14 % preferred clinic retesting (Table 2). Among home-based retesters who said they preferred home testing in the future, the main reasons were that it saves time (70 % *n* = 64) and was more convenient (57 %, *n* = 52), followed by more confidential (27 %, *n* = 25) and less embarrassing (22 %, *n* = 20). For those who preferred clinic retesting (*n* = 20), the main reasons were that they were more confident that the tests would be done properly (65 %, *n* = 13), followed by more confidential (30 %, *n* = 6), more convenient (20 %, *n* = 4), and would like to be able to talk to a clinician (10 %, *n* = 2).

Comparing those with a preference for home testing to those who didn’t, there were significant differences in being comfortable having the kit sent to their home (*p* = 0.045), living with friends/flatmates rather than with their partner, parents or alone (*p* = 0.034) and not being diagnosed with chlamydia previously (*p* = 0.030). Of those who preferred home testing, 62 % lived with their friends/ flatmates, 16 % alone, 11 % with a partner and 10 % with parents, compared with 37 %, 27 %, 18 % and 12 % respectively among those who preferred clinic retesting or didn’t have a preference (Table 2).

#### Preference for receiving kit (home arm participants only)

The majority of home-based retesters (91 %, *n* = 129) said they would prefer to be sent their kit in the mail and 9 %, (*n* = 12) said they preferred to collect their kit from the clinic. There were no significant differences in preference for receiving kits by age group (≤25 years or > 25 years), risk group or who they lived with (Table 3).

**Table 3:** Preferred method of receiving home test kits by age group, risk group and living arrangements

		Prefer mail	Prefer to collect	Total	p-value^a^
		n (%)	n (%)	n (%)	
Study arm	Home	129 (91.5)	12 (8.5)	141 (100)	
Age group	≤25	54 (41.9)	5 (41.7)	59 (41.8)	0.632
	>25	73 (56.6)	7 (58.3)	80 (56.7)	
Risk group	Women	47 (36.4)	5 (41.7)	52 (36.9)	0.469
	Heterosexual men	38 (29.5)	5 (41.7)	43 (30.5)	
	MSM	44 (34.1)	2 (16.7)	46 (32.6)	
Who they live with?	Friends/flatmates	70 (54.3)	5 (41.7)	75 (53.2)	0.409
	Alone	27 (20.9)	1 (8.3)	28 (19.9)	
	Partner	15 (11.6)	4 (33.3)	19 (13.5)	
	Parents	13 (10.1)	2 (16.7)	15 (10.6)	
	Other	4 (3.1)	0 (0.0)	4 (2.8)	

*MSM* men who have sex with men

^a^Chi^2^ or Fisher’s exact test was used when appropriate

### Cost

#### Cost of the clinic retest pathway per person

The overall cost per person of the clinical pathway from initial testing to result notification, treatment visit and retesting at the clinic was $168.60 (MSM $216.30, heterosexual men $142.50, women $145.30): $80.10 for the initial consultation, $3.70 for result notification and SMS, $14.90 for the treatment visit, and $69.90 for the retest visit (Table 4). Overall costs were greater for MSM due to the additional cost of testing two samples (rectal swab and urine), versus a single sample for heterosexual men (urine) and women (vaginal swab).

**Table 4:** Costs (AUD) per study step per person

	Clinic retest				Home retest			
Study step	MSM	HM	W	Total^a^	MSM	HM	W	Total^a^
Initial consultation								
Registration								
New	3.9	3.9	3.9	3.9	3.9	3.9	3.9	3.9
Existing	2.4	2.4	2.4	2.4	2.4	2.4	2.4	2.4
Consultation	24.7	23.9	25.2	24.7	24.7	23.9	25.2	24.7
Laboratory fee	71.8	35.9	35.9	47.9	71.8	35.9	35.9	47.9
Antibiotics^b^	1.2	1.2	1.2	1.2	1.2	1.2	1.2	1.2
Result notification								
Staff time	3.6	3.6	3.6	3.6	3.6	3.6	3.6	3.6
Phone call	0.1	0.1	0.1	0.1	0.1	0.1	0.1	0.1
Treatment^b^								
Registration	1.4	1.4	1.4	1.4	1.4	1.4	1.4	1.4
Consultation	12.0	11.1	11.3	11.7	12.0	11.1	11.3	11.7
Antibiotics	1.7	1.7	1.7	1.7	1.7	1.7	1.7	1.7
SMS	0.1	0.1	0.1	0.1	0.1	0.1	0.1	0.1
Retest								
Registration	2.0	2.0	2.0	2.0	N/A	N/A	N/A	N/A
Assembly of home test kits	N/A	N/A	N/A	N/A	3.4	3.4	3.4	3.4
Consultation	19.4	19.1	20.6	19.8	N/A	N/A	N/A	N/A
Equipment^c^	0.2	0.2	0.1	0.2	1.5	0.9	0.9	1.1
Postage^d^	N/A	N/A	N/A	N/A	13.2	13.2	13.2	13.2
Laboratory fee	71.8	35.9	35.9	47.9	55.4	28.7	28.7	37.6
Sub-total retest component only	93.4	57.2	58.6	69.9	73.5	46.2	46.0	55.3
Total^a^	216.3	142.5	145.3	168.6	196.40	131.5	133.0	154.0

*MSM* men who have sex with men, *HM* heterosexual men, *W* women, *N/A* not applicable

^a^All costs expressed in Australian dollars (AUD) (2012)

^b^Based on 40 % treated at consult therefore only 60 % needing a treatment visit

^c^For clinic retesters clinics supplied urine jars (men) and pathology forms and for home retesters, postage satchels and saline for rectal swabs (MSM) were provided by the study. All other testing equipment was supplied by the laboratories and included in the laboratory fee

^d^Includes return postage

#### Cost of the home retesting strategy per person

The home retesting strategy included the costs for the initial testing, result notification and treatment visit at the clinic, plus the cost of retesting at home. The overall cost per person of this strategy was $154.00 (MSM $196.40, heterosexual men $131.50, women $133.00); $80.10 for the initial consultation, $3.70 for result notification and SMS, $14.90 for the treatment visit, and $55.30 for the retesting component (Table 4).

#### Cost per infection

In the home arm, 61 % (*n* = 184) of participants retested. Of these, all were sent home testing kits, and 73 % (*n* = 134) self-collected at home and mailed their specimen/s to the laboratory and 27 % (*n* = 50) retested at the clinic. In total, 31 repeat infections were detected. In the clinic arm, 39 % (*n* = 117) retested at the clinic and 12 repeat infections were detected. Based on these results, the overall cost per repeat infection detected was estimated to be $1409.20 for the home retesting strategy and $3132.60 for the clinic retesting pathway: a difference of $1723.40 per infection detected (Table 5).

**Table 5:** Cost per infection of home and clinic retesting

Trial arm	Retest uptake	n	%	Cost of retest component $	Cost of overall retest pathway $	Overall cost $	No. of repeat infections	Cost per repeat infection $
Clinic retest	No retest	181	61		98.7^a^	17864.7		
	Retest	117	39	69.9	168.6	19726.2		
	Total	298				37590.9	12	3132.6
Home retest REACT study	No retest	118	39		116.4^b^	13735.2		
	Retest at home	134	44	55.3	154.0	20636.0		
	Sent home kit but retested at the clinic	50	17		186.3^c^	9315.0		
	Total	302				43686.2	31	1409.2

^a^Includes all costs in the clinic retest pathway except the retest component

^b^Includes all costs in the home retest strategy except the laboratory fee

^c^Includes all costs in the home retest strategy except the laboratory fee, plus the cost of the clinic retest component

## Discussion

Home-based retesting and SMS reminders were found to be acceptable and home-based retesting was preferred to clinic-based retesting by the majority of home arm retesters. However 14 % of home arm retesters preferred clinic-based retesting, which may relate to confidentiality concerns in their home environment. Evaluation of costs showed that the home retesting strategy was cost saving compared to clinic-based retesting ($154 vs $169). The cost saving became more pronounced when examining the cost per infection detected due to the effectiveness of the home-based strategy in detecting more repeat infections (31 vs 12). The cost per reinfection detected via the home based strategy was $1409 per reinfection detected compared with $3133 for the clinic-based strategy.

Home-based STI screening with self-collected specimens has been shown to be feasible and acceptable in both men and women [28]. In our study, most participants preferred home-based retesting to clinic-based retesting with the main reasons relating to convenience. These findings are consistent with other studies [29, 30]. However there was a small subset of participants who preferred clinic-based retesting, particularly participants who lived with their partner, parents or alone. For those who lived with partners or parents, this may have been due to concerns about confidentiality. Another Australian study found that young people were less likely to request a home-collection kit if they lived with their parents [31]. For those living alone, concerns may have related to the size of the home test kit which was too large for an average letter box, and the inconvenience of having to collect it from the post office if no-one was home when it was delivered. In our survey, participants were also less likely to prefer home testing if they had a previous chlamydia diagnosis. This group may have developed a rapport with the clinic staff and a feeling of trust in the clinic. As has been demonstrated in other studies [21, 25, 31], a person’s social circumstances or experiences are likely to be key factors in their retesting preference, and providing options for retesting is therefore important. Another important consideration is that MSM should be offered repeat testing for other infections including syphilis and HIV. An option may be to ask MSM to return to the clinic for retesting and if they don’t return, to send them a home test kit.

There is very limited evidence about the cost of home versus clinic based retesting. In the context of the DAISY study undertaken in the USA, where young women were randomised to an intervention group to receive home testing kits for chlamydia and gonorrhoea by mail at 6, 12 and 18 months, or a control group who received a postcard at the same intervals inviting them to attend one of the participating study clinics at no cost, Smith and colleagues compared the direct and indirect costs of home and clinic based screening [32]. It was found that home collection was cost saving overall (25 USD per home test versus $111 per clinic test, including $49 in direct costs and $62 in indirect costs such as transportation and parking, child care and missed work (2005 prices) [32]. The cost savings were greater in this study as indirect costs to the patient were included, and also the purpose of the home-test component was for screening (not retesting) and did not include any additional interactions with the clinic.

In the REACT trial, taking into consideration the costs of the entire pathway, home retesting was cost saving overall at $154 per test versus $169 for clinic based retesting. The two key differences in costs between the strategies were: the cost of the retesting consultation in the clinic pathway which was slightly higher than the cost of assembling home test kits plus postage; and the laboratory fee in the clinic pathway was greater than the home strategy as a different laboratory was used. Had the laboratory costs been equal, the cost of home retesting would still have been less costly but only marginally so. The home-testing cost was likely to be an over-estimation as we conservatively included 5 min time for assembly of kits which included identifying those patients who were due to be sent a home kit and organising the kit to be mailed. If kits were pre-assembled by the laboratory and identification of patients was automated, then clinic costs would be lower. The cost of a routine program depends on how it is implemented, for example if kits were only sent to those who requested them or had not returned to the clinic, it may be less costly, but the extra complexity would need to be considered.

In the REACT trial, more infections were detected in the home arm compared to the clinic arm. The effectiveness of the home retesting strategy in detecting more repeat infections, particularly among MSM, has the potential for considerable downline cost savings to the health system through the reduction of onward transmission and reducing the risk of sequelae including HIV transmission. The cost per infection detected in the REACT trial was considerably less at $1409 for home testing versus $3133 for clinic testing, mainly due to the higher number of repeat infections detected via the home based strategy (31 vs 12). Modelling by Smith and colleagues [32] in the DAISY trial also found the cost per infection to be slightly less overall ($702 in the home arm versus $717 in the clinic arm), as fewer clinic based tests were performed (3.6 tests per person in the intervention arm versus 2.7 tests per person in the clinic arm) [32], but the difference was less than REACT as the infection rate was equal in the two arms. A strength of the study was the high survey response rate. There are some limitations to consider. We didn’t estimate the cost to the patient (indirect costs). As shown by Smith et al. in the DAISY trial, inclusion of indirect costs such as transportation/ parking, child care costs and time missed from work/ school, would probably have doubled the costs of the clinic-based retesting arm in REACT, hence cost savings for home retesting would have been greater. It is also important to consider the generalisability of these findings. Costs associated with clinic-based retesting in this study related to a sexual health clinic where patients were seen mainly by nurses. However in primary care, patients would be more likely to see a doctor with a higher salary and thus there would be even greater cost savings for the home-testing strategy in this setting. Acceptability may have been over-estimated as those who chose to respond to the survey may have been more likely to give positive responses. In addition, participants were not blinded to their study arm allocation which may have had a differential impact on the likelihood of them retesting, however there is no way to assess this. Also as the study was conducted in the sexual health clinic setting, the results may not necessarily apply to other primary care settings.

## Conclusions

In conclusion, the study has shown that in the sexual health clinic setting, a multi-faceted intervention previously shown to be effective at increasing retesting rates, was also acceptable to patients, and cost-efficient. However some clients prefer clinic-based testing, often due to confidentiality concerns in their home environment. Both options should be provided to maximise retesting rates.

## Additional file

Additional file 1:
**REACT Survey.** (PDF 379 kb)

## Abbreviations

AUD: Australian dollars
HIV: human immunodeficiency virus
HREC: human research ethics committee
MSM: men who have sex with men
REACT: retest after *Chlamydia trachomatis*
SMS: short message service
STI: sexually transmissible infection
