# Supplementary material for: The acceptability and cost of a home-based chlamydia retesting strategy: findings from the REACT randomised controlled trial
Source: BMC Public Health. 2016 Jan 28;16:83. doi: 10.1186/s12889-016-2727-4 (PMC4730759; doi:10.1186/s12889-016-2727-4)
Supplement: Additional file 1: — REACT Survey. (PDF 379 kb) [file 12889_2016_2727_MOESM1_ESM.pdf]

react

Chlamydia Re-test Trial

Questionnaire

All information you provide will be treated as strictly confidential. No reference to your name or contact details will appear anywhere on this questionnaire. You will be reimbursed for your time in completing this questionnaire with a $40 Coles/ Myer voucher.

Please enter your study code ____________

Part A

We would like to ask you about your age and your living situation

1 What is your gender?

☐ Male ☐ Female

2 What age group do you belong to?

☐ 16 – 25 ☐ 26 – 35 ☐ 36 – 45 ☐ 46 – 55 ☐ Over 55

3 Who do you currently live with?
Tick one or more options

☐ Partner ☐ Friends / flatmates ☐ Parents ☐ Alone
☐ Other *(please specify)* ____________________

Part B

We would like to ask you some personal questions about your sexual relationships.

4 When you were diagnosed with chlamydia at the start of the study, did you take the antibiotic/s given to you by the sexual health clinic?

☐ Yes – all of them ☐ Yes – some of them ☐ No

5 Since being diagnosed with chlamydia at the start of the study, have you had sex?

☐ Yes ☐ No *– skip to question 11 for women or 15 for men*

6 Since you were treated for chlamydia at the start of the study, did you use a condom every time with each partner you had sex with?

☐ Yes *– skip to question 11 for women or 15 for men* ☐ No ☐ Not sure

7 <u>Of the partners you didn't</u> always use condoms with, were they:

☐ A new sexual partner/s (you had sex with them for the first time since you were treated for chlamydia) *– skip to question 10*
☐ An existing partner/s (you had sex with them prior to being treated for chlamydia)
☐ You had sex with both new and existing partners

8 As far as you know, were <u>all</u> your existing partners in the last 6 months treated for chlamydia?

☐ Yes ☐ No *– skip to question 11for women or 15 for men* ☐ Don't Know *– skip to question 11 for women or 15 for men*

Part B continued

9 **After you were treated for chlamydia , did you have sex with your existing partner/s before they were treated?**

- ☐ Yes
- ☐ No
- ☐ Don't Know

10 **Since you were treated for chlamydia at the start of the study, have any of your sexual partners had sex with other people at around the same time they had sex with you?**

- ☐ Yes / I think so
- ☐ No / I don't think so
- ☐ Not sure

Male participants- skip to question 15

Part C

FEMALE PARTICIPANTS ONLY:
We would like to ask you about symptoms which might be pelvic inflammatory disease (PID).

11 **Have you had any of the following symptoms since you were treated for chlamydia at the start of the study:**
Tick any that apply

- ☐ Lower abdominal pain
- ☐ Bleeding after sex
- ☐ Lower abdominal pain with sex
- ☐ Abnormal vaginal discharge (change in usual amount or colour)
- ☐ Bleeding between periods
- ☐ None of the above *– skip to question 15*

If yes to any of the above, do you agree to being contacted by a member of the research team to discuss your symptoms in more detail to decide if they might be symptoms of PID?

- ☐ Yes
- ☐ No

12 **How long have you had these symptoms?**

- ☐ Less than 1 month
- ☐ Between 1 & 3 months
- ☐ Between 3 & 12 months
- ☐ More than 12 months

13 **Did you see a doctor/nurse about these symptoms?**

- ☐ Yes - at the **same** sexual health clinic where I was tested for chlamydia at the start of the study
- ☐ Yes at **another** clinic
- ☐ Yes at a hospital
- ☐ No

14 **If you attended another clinic or hospital**

a. What was the name of the clinic or hospital ____________________

b. Approximately when did you attend ____________________

c. Do you give us permission to contact your doctor to get a copy of the relevant medical records? These will be kept confidential.

- ☐ Yes
- ☐ No *If yes, we will send you a consent form for this information*

Part D

Chlamydia tests can be done in a number of different ways. We are interested to know how you were tested for chlamydia and how you prefer to be tested.

15 **When you were tested for chlamydia at the start of the study, how were you tested?**

- ☐ Specimens were collected by a doctor/nurse at the clinic
- ☐ I collected my own specimen/s at the clinic

16 **Since your first test at the start of the study, have you had a follow-up chlamydia test (i.e another test since your positive test)?**

- ☐ Yes – at the same sexual health clinic
- ☐ Yes – at another clinic
- ☐ Yes – I mailed a specimen that I collected at home *– skip to question 20*
- ☐ No *– skip to question 19*

17 **How were you tested when you had your follow-up test?**

- ☐ Specimens were collected by a doctor/nurse at the clinic
- ☐ I collected my own specimen(s) at the clinic

18 **If you were tested at another clinic:**

a. What was the name of this clinic? ______________________

b. Approximately when did you have the test? ______________________

c. Do you give us permission to contact this clinic to get a copy of the result? The result will be kept confidential

☐ Yes ☐ No *If yes, we will send you a consent form for this information*

19 **If you did not have a follow-up test, could you tell us why you didn't have another chlamydia test?**
Please tick one or more options

- ☐ I didn't think I was at risk
- ☐ I didn't have time
- ☐ I misplaced my kit
- ☐ Other *(please specify)* ______________________
- ☐ I wasn't sure how to test myself
- ☐ I didn't receive my kit in the mail

Part E

These questions only apply to those who **collected their own follow-up specimen/s at home.**
If you had your follow-up test at a clinic, skip to question 29.

20 **How comfortable did you feel having the kit posted to your home?**

- ☐ Very comfortable
- ☐ Comfortable
- ☐ Neutral
- ☐ Uncomfortable
- ☐ Very uncomfortable

Please explain ______________________

____

Part E continued

21 How easy to understand were the instructions that were provided with the kits?

- ☐ Very easy
- ☐ Easy
- ☐ Neutral
- ☐ Hard
- ☐ Very hard
- ☐ Didn't use

Please explain ____________________

____

22 How easy was it to collect your own specimen/s?

- ☐ Very easy
- ☐ Easy
- ☐ Neutral
- ☐ Hard
- ☐ Very hard

Please explain ____________________

____

23 How confident were you that you collected the specimens correctly?

- ☐ Very confident
- ☐ Reasonably confident
- ☐ Neutral
- ☐ Not very confident
- ☐ Not confident at all

Please explain ____________________

____

24 Were there any problems collecting the specimens? *Please tick one or more options*

- ☐ Urine splashed on hands
- ☐ Wasn't sure where the swab should go
- ☐ Wasn't sure how far to insert swab
- ☐ Difficult to aim the urine onto the swab
- ☐ Wasn't sure how far to insert the swab
- ☐ None of the above
- ☐ Other, *please specify* ____________________

____

25 Approximately how many days after you collected your specimen/s did you mail them to the lab? ____________

26 Which do you prefer, having the test in the clinic, or collecting your own specimens at home?

- ☐ Testing at the clinic
- ☐ Testing at home
- ☐ Neutral

27 If you prefer testing at home or the clinic, why do you prefer that method? *Please tick one or more options*

- ☐ Less embarrassing
- ☐ More convenient
- ☐ Saves time
- ☐ More confidential
- ☐ More confident test will be done properly
- ☐ Other, *please specify* ____________________

____

Part E continued

28 **How would you prefer to receive a home testing kit?**

- ☐ In the mail
- ☐ Pick up at clinic
- ☐ Other, *please specify* ____________________

Part F

We would like to know how you prefer to be reminded about having a follow-up test.

29 **How did you feel about receiving the SMS reminders to have a follow-up test?**

- ☐ Very comfortable
- ☐ Comfortable
- ☐ Neutral
- ☐ Uncomfortable
- ☐ Very uncomfortable

Please explain ____________________

30 **Could the wording and/or timing of the SMS reminders be improved?**

- ☐ Yes
- ☐ No
- ☐ Not sure

Please explain ____________________

31 **How would you prefer to be reminded about having a follow-up test?**

- ☐ SMS (text message)
- ☐ Email
- ☐ Phone call
- ☐ Letter
- ☐ Not at all
- ☐ Other, *please specify* ____________________

Thank you very much for your time in completing this questionnaire.

We will be posting a Coles/Myer voucher to you shortly. If you have changed address, please let us know your new address by phoning 1800330712.

Please mail the completed questionnaire to:

Dr. R. Guy
The Kirby Institute
University of NSW
Cliffbrook Campus,
45 Beach St,
Coogee, NSW 2031
